# Caspase-1 and Cathepsin B Inhibitors from Marine Invertebrates, Aiming at a Reduction in Neuroinflammation

**DOI:** 10.3390/md20100614

**Published:** 2022-09-29

**Authors:** Rafaela Indalecio Moreno, Vanessa O. Zambelli, Gisele Picolo, Yara Cury, André C. Morandini, Antonio Carlos Marques, Juliana Mozer Sciani

**Affiliations:** 1 Laboratório Multidisciplinar de Pesquisa, Universidade São Francisco, Bragança Paulista 12916-900, Brazil; 2 Unidade Integrada de Farmacologia e Gastroenterologia (UNIFAG), Bragança Paulista 12916-900, Brazil; 3 Laboratório de Dor e Sinalização, Instituto Butantan, São Paulo 05503-900, Brazil; 4 Departamento de Zoologia, Instituto de Biociências, Universidade de São Paulo, São Paulo 05508-090, Brazil; 5 Centro de Biologia Marinha, Universidade de São Paulo, São Sebastião 11612-109, Brazil

**Keywords:** caspase-1, cathepsin B, inhibitor, Cnidaria, Echinodermata, neuroinflammation, trigonelline, betaine

## Abstract

Neuroinflammation is a condition associated with several types of dementia, such as Alzheimer’s disease (AD), mainly caused by an inflammatory response to amyloid peptides that induce microglial activation, with subsequent cytokine release. Neuronal caspase-1 from inflammasome and cathepsin B are key enzymes mediating neuroinflammation in AD, therefore, revealing new molecules to modulate these enzymes may be an interesting approach to treat neurodegenerative diseases. In this study, we searched for new caspase-1 and cathepsin B inhibitors from five species of Brazilian marine invertebrates (four cnidarians and one echinoderm). The results show that the extract of the box jellyfish *Chiropsalmus quadrumanus* inhibits caspase-1. This extract was fractionated, and the products monitored for their inhibitory activity, until the obtention of a pure molecule, which was identified as trigonelline by mass spectrometry. Moreover, four extracts inhibit cathepsin B, and *Exaiptasia diaphana* was selected for subsequent fractionation and characterization, resulting in the identification of betaine as being responsible for the inhibitory action. Both molecules are already found in marine organisms, however, this is the first study showing a potent inhibitory effect on caspase-1 and cathepsin B activities. Therefore, these new prototypes can be considered for the enzyme inhibition and subsequent control of the neuroinflammation.

## 1. Introduction

Neuroinflammation is defined as an inflammatory response that occurs in the brain or spinal cord, mediated by the production and release of cytokines, chemokines, reactive oxygen species, and secondary messengers [1]. Inflammatory mediators are centrally produced by resident central nervous system (CNS) glia (microglia and astrocytes), endothelial cells, and peripherally derived immune cells.

The release of pro-inflammatory cytokines is reported in several diseases, such as Alzheimer’s disease (AD), the main type of dementia, and the degree of neuroinflammation depends on the context, duration, and course of the primary stimulus or injury [1,2]. Recent research points to the participation of cathepsin B and caspase-1 from inflammasomes in this process [3].

Inflammasomes are cytosolic protein complexes, assembled after activation by Aβ peptides from AD, whose main mechanism is the activation of the NLRP3 pathway in microglia. After binding to NOD-like membrane receptors, a central domain, NATCH, favors receptor oligomerization, allowing the interaction with a caspase recruitment domain (CARD) or a pyrin domain (PYD), recruiting pro-caspases [4]. The inflammasome protein 3, containing a C-terminal portion rich in NOD-leucine repeats and pyrin domain (NLRP3), which, under normal conditions, is maintained in its inactive form in the endoplasmic reticulum and the ASC located in the mitochondria. When activated, the interaction between NLRP3 and ASC occurs by the polymerization of PYD and ASC filaments, and this complex leads to the recruitment of pro-caspase-1 via CARD, leading to cleavage and activation, and the subsequent release of caspase-1, which, in turn, activate nuclear factor-κB (NF-κB), increasing the expression of pro-IL-1β and pro-IL-18 cytokines [5,6].

The NLRP3 inflammasome was co-localized with amyloid plaques in patients with AD. It is seen that the Tau protein is also able to activate NLRP3 and the inflammasome formation pathway [6]. Experiments using caspase-1, APP, PS1, and NLRP3 knockout mice demonstrate that inflammation mediated by the NLRP3, and caspase-1 pathway contribute to AD cognitive and behavioral dysfunction [7,8]. Thus, it is believed that therapies that cause the inhibition of inflammasome formation may contribute to the reduction in AD progression.

The production of dysfunctional and toxic proteins/peptides that are the cause of other types of dementia, such as aggregated α-synuclein from Parkinson’s disease or huntingtin from Huntington’s disease, are able to activate caspase-1 through inflammasome assembly [9,10].

In addition to activating inflammasome, oligomeric Aβ from AD also activates cathepsin B, and then induces the production of reactive oxygen species (ROS). Furthermore, Aβ induces the cathepsin-B-mediated activation of the NLRP3 inflammasome to initiate processing of pro-caspase-1 to caspase-1, and the subsequent conversion of pro-IL-1β to active inflammatory factor IL-1β. Thus, these two enzymes share a common cytokine release pathway [11].

Cathepsin B are mammalian cysteinopeptidases, present in most cells and tissues. They are lysosomal enzymes that act on the intracellular degradation of proteins, but they can act extracellularly when released under certain circumstances, degrading components of the extracellular matrix [12].

In general, cathepsins B are involved in various physiological and pathophysiological processes, such as cell cycle regulation, cancer development, autophagy, and neuroinflammation [13]. The enzyme is also involved in several diseases of the central nervous system, mainly in AD [14,15,16,17,18].

In AD, the intracellular formation of amyloid peptides causes disruption in the membrane of lysosomes, which leads to leakage of the cathepsin B from the compartment into the cytosol. In humans with AD, the concentration of cathepsin B is increased in the cytoplasm, in contrast to its location in lysosomes, and the enzyme is redistributed in the pathology [19]. The cytosolic enzyme then causes caspase-dependent cell death by cleaving the anti-apoptotic protein Bcl-2 and removing the apoptosis-preventing protein, Bcl-xl, causing cell death [11].

Overexpression of cathepsins B, L, and X is reported in AD [20]. Many studies found high levels of cathepsin B, or its increased activity, in plasma and cerebrospinal fluid [21]. Furthermore, increased plasma levels of cathepsin B are related to cognitive dysfunction of AD [22].

In animal models genetically modified to express the mutated APP that causes AD (APP KM670/671NL, Swedish; APP (716V, Florida; APP V171I, London)), cathepsin B concentration was evaluated at the protein and gene levels, and are increased ~50% in the cortex and hippocampus compared to a control group without the mutation [23]. When the gene that expresses cathepsin is knocked out, there is an improvement in memory deficits, which is observed in animals with AD-like symptoms [18].

Thus, obtaining new inhibitors of such enzymes (caspase-1 and cathepsin B), which are proven to be important for the development of neuroinflammation, may be an alternative to the AD treatment.

In this sense, natural products can provide new molecular entities, especially marine animals, which are poorly explored from the biochemical and pharmaceutical point of view. Therefore, the discovery of new molecules could result in new prototypes that could contribute to a reduction in neuroinflammation, and be an auxiliary treatment for neurodegenerative diseases.

## 2. Results

**Figure 1:**
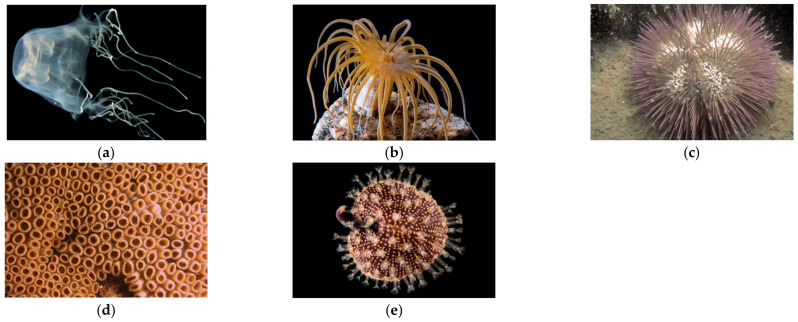
Photo of the five species collected, in which their extracts were fused in the study. (**a**) *Chiropsalmus quadrumanus*, (**b**) *Exaiptasia diaphana*, (**c**) *Lytechinus variegatus*, (**d**) *Palythoa caribaeorum*, (**e**) *Renilla reniformis*. Photographs by Alvaro Migotto (University of São Paulo).

**Figure 2:**
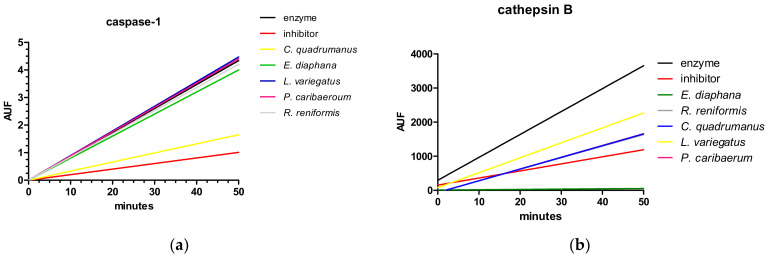
Enzymatic assay to identify inhibitory effect of 5 marine extracts on caspase-1 (**a**) and cathepsin B (**b**) as arbitrary units of fluorescence (AUF) over time. The black line represents the enzyme and the red line the known inhibitors (Ac-YVAD-CHO for caspase-1, and F-F-FMK for cathepsin B). Extracts were obtained from four cnidarians (*Exaiptasia diaphana*, *Chiropsalmus quadrumanus*, *Palythoa caribaeorum*, *Renilla reniformis*) and one echinoderm (*Lytechinus variegatus*).

Extracts from five marine invertebrates belonging to two different phyla (Cnidaria and Echinodermata), shown in Figure 1, were tested to verify the putative capacity to inhibit caspase-1 (Figure 2a) and cathepsin B activity (Figure 2b). The experiment was carried out in a simultaneous evaluation in direct comparison to a known inhibitor for the correspondent target enzyme. *Chiropsalmus quadrumanus* extract is the only one able to inhibit caspase-1, and four extracts (*Exaiptasia diaphana, C. quadrumanus*, *Renilla reniformis*, and *Palythoa caribaeorum*) inhibit cathepsin B activity, but only *E. diaphana* inhibits more than the known inhibitor, F-F-FMK.

The extracts with best performance for both enzymes (*C. quadrumanus* and *Exaiptasia diaphana*) were selected to be fractionated and tested again, in order to find the active molecule to be characterized. These results are shown in the subsequent sections.

### 2.1. Caspase-1

**Figure 3:**
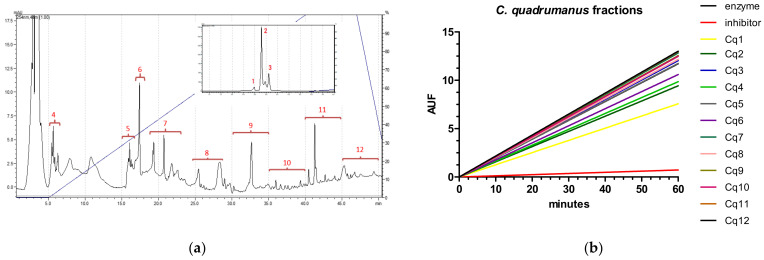

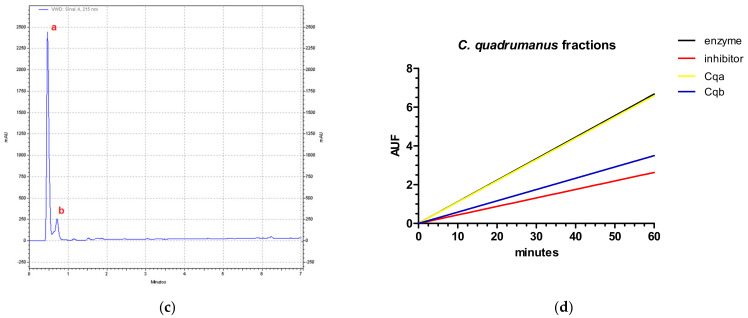
Fractioning and enzymatic assay of *Chiropsalmus quadrumanus* extract. (**a**) Chromatogram of *C. quadrumanus* extract obtained in a C18 column coupled to a HPLC, set for molecules fractionation. Numbers indicate the 12 collected fractions. (**b**) Caspase-1 enzymatic assay of *C. quadrumanus* fractions, shown as arbitrary units of fluorescence (AUF) over time (in minutes). The black line represents enzyme activity, the red line represents the enzyme incubated with Ac-YVAD-CHO inhibitor and others represent the enzyme incubated with one of the *C. quadrumanus* fractions, obtained by HPLC. (**c**) Re-fractionation of Cq1, the fraction with better inhibitory activity on caspase-1, yielding 2 fractions, named Cqa and Cqb. (**d**) Enzymatic assay of fractions Cqa and Cqb to test their inhibitory activity on caspase-1. The black line represents the enzyme, the red line represents the enzyme incubated with Ac-YVAD-CHO inhibitor, the yellow line is the fraction Cqa, and the blue line represents the enzyme incubated with Cqb fraction.

The *C. quadrumanus* extract was chosen for HPLC fractionation due to its high caspase-1 inhibitory activity. The extract was separated into 12 fractions (Figure 3a) and each fraction was individually tested again for caspase-1 activity. The results led us to select the fraction (Cq1) due to its reduced velocity (enzyme = 0.26756 AUF/min, inhibitor = 0.015771 AUF/min, Cq1 = 0.12262 AUF/min), as shown in Figure 3b.

The Cq1 fraction was analyzed by mass spectrometry and impurities were verified (data not shown). Thus, a new fractionation step was performed (Figure 3c), yielding two fractions, named a and b. One of them (Cqb) was identified as the active one (Figure 3d), and its activity is compared to the known inhibitor.

**Figure 4:**
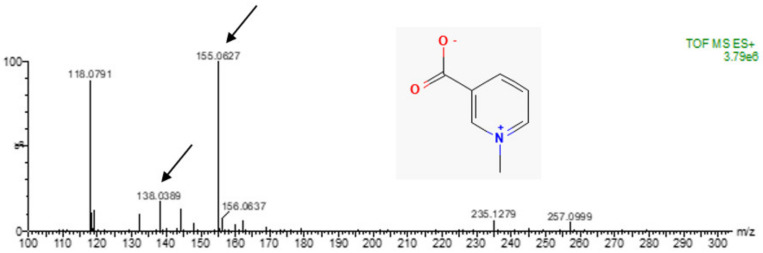
Mass spectra of the isolated molecule from *Chiropsalmus quadrumanus*, obtained after HPLC fractionation, with inhibitory effect on caspase-1. The arrows show the ion (138.0389 *m/z*) and the adduct (155.0627) corresponding to trigonelline. The detail inserted represent the trigonelline structure identified by database spectra comparison.

This molecule, Cqb, was analyzed by mass spectrometry, as shown in Figure 4. The ion 118.0791 is observed in other fractions, even those not active in inhibiting caspase-1. Thus, the 155.0627 *m/z* was chosen for molecule identification. By comparing to databases, trigonelline (C_7_H_7_NO_2_) is identified with highest score, with 202 ppm error, being identified with its neutral mass and adducts.

### 2.2. Cathepsin B

**Figure 5:**
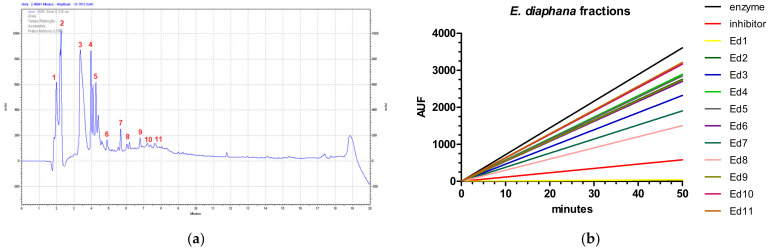
Fractioning and enzymatic assay of *Exaiptasia diaphana* extract. (**a**) Chromatogram obtained in a C18 column coupled to a HPLC, used in the molecules fractionation. Numbers indicate the 11 collected fractions. (**b**) Cathepsin B enzymatic assay of *E. diaphana* fractions, shown as arbitrary units of fluorescence (AUF) over time (in minutes). The black line represents enzyme activity, the red line represents the enzyme incubated with F-F-FMK inhibitor, and other lines represent enzymes incubated with each *E. diaphana* fraction.

The fractioning process of *E. diaphana* extract yields 11 fractions (Figure 5a). Each fraction was then lyophilized and tested again for cathepsin B inhibitory activity. It is verified that fraction 1 is efficient in inhibiting the enzyme (yellow line in Figure 5b, velocity 0.1 AUF/min)), as well as the known inhibitor (red line, velocity 1.08 AUF/min), compared to the enzyme without any treatment (black line, velocity 46.57 AUF/min).

**Figure 6:**
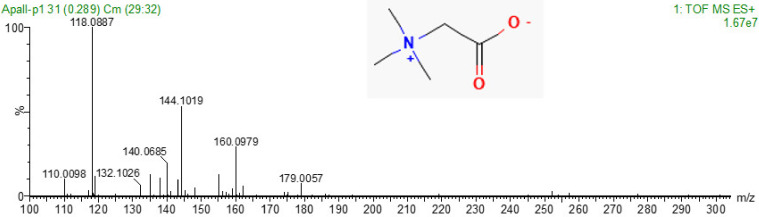
Mass spectra of the isolated molecule from *Exaiptasia diaphana*, obtained after HPLC fractionation, with inhibitory effect on cathepsin B. The detail inserted represents the betaine structure, identified by database spectra comparison.

The inhibitory fraction (named Ed1) was analyzed by mass spectrometry (Figure 6), and the ion 118.0887 *m/z* is found as the most abundant. This ion was submitted to an analysis in small molecules databases, and a high correlation with betaine (or N,N,N-trimethylglycine) is found, with 2.96 ppm error.

## 3. Discussion

Neuroinflammation is a characteristic of several types of brain diseases, caused by the release of inflammatory mediators, [7]. In this scenario, we considered two molecular targets in this study, viz., caspase-1 and cathepsin B, which are applicable to several brain diseases involving inflammatory effect, but important targets for Alzheimer’s disease (AD), considering that, in the pathology, both enzymes are activated by amyloid peptides and cause effects on neurodegeneration [21]. In other dementia types, such as PD, the cathepsin B rule is controversy, but it is believed that the enzyme is important for protein clearance, so its inhibition would be prejudicial for the patients [24].

A high level of activity of the active enzyme caspase-1 has already been observed in the brains of patients with AD compared to patients without the disease, a result consistent with the activation of the inflammasome [7,25]. Although some caspase-1 inhibitors have been studied, few studies focus on AD. The VX-765 inhibitor was studied in mice, and reduces episodic memory impairment in a dose-dependent manner, at the same time that it reduces the deposition of beta-amyloid peptides and decreases neuroinflammation [26].

In a multiple sclerosis model, VX-765 reduces inflammasome assembly in the central nervous system, preventing axon injury, with consequent improvement in neurobehavior performance [27].

To the best of our knowledge, this is the first time that molecules extracted from marine invertebrates have shown caspase-1 inhibitory effects. This study demonstrates the presence of a molecule from a box jellyfish (*C. quadrumanus*) with an inhibitory effect on such enzymes. This species is still little studied from a biochemical and bioprospecting perspectives.

We studied the putative actions of molecules of *C. quadrumanus* in neurons under several aspects. We demonstrate that different concentrations of the methanolic extract have no toxic effect on the differentiated human neuroblastoma SH-SY5Y cell line. It is found that the extract causes neurite elongation and branch formation, while not affecting cell proliferation, necrosis, or apoptosis [28]. These data indicate that there are potential molecules that could be used to increase neuron connection, useful for neurodegenerative diseases such as Alzheimer’s. Added to this network improvement, the extract may have the potential to reverse neuroinflammation, as demonstrated here, by inhibiting caspase-1 and, consequently, inhibiting inflammasome formation and the release of pro-inflammatory cytokines.

The *C. quadrumanus* caspase-1 inhibitor identified in this study is trigonelline, which is an alkaloid from the pyridine group, commonly found in plants, with the known property of lowering blood glucose [29]. This compound has already been found in marine animals, *viz.*, the sea anemone *Anemonia sulcata* [30], the sea urchin *Arbacia lixula* (as *Arbacia pustulosa*), and the pleustonic polymorphic colony *Velella velella* (as *Velella spirans*) [31]. Trigonelline is known to act by antagonizing the induction of larval metamorphosis in both hydrozoans (*Hydractinia* and *Eirene*) and scyphozoans (semaeostomes *Chrysaora hysoscella* and *Cyanea lamarckii,* and the rhizostome *Cassiopea* spp.) [32,33], and defense molecule against predators in the sponge *Xestospongia* sp. [34].

Trigonelline has many physiological effects demonstrated: it is able to inhibit mice caspase-3, protect pancreatic beta cells from apoptosis, induces anti-inflammatory effect with a reduction in IL-6 and IL-1β, and acts to increase the antioxidant enzymes activity [35]. In addition to the anti-inflammatory effect, the percentage of axons and the size of dendrites increases in the mice’s nervous systems [36], a pattern also observed in a previous study of the action of *C. quadrumanus* extract [28]. Finally, an effect on the inhibition of acetylcholinesterase activity, a target of current AD treatment, is also reported [37].

In addition to the caspase-1 inhibitor, we also found an inhibitor molecule for cathepsin B. Cathepsin B is correlated with inflammasome assembly, and cytosolic cathepsin B, resulting from lysosomes disruption after amyloid peptide action, causes caspase-1 activation [38]. Although it is shown that cathepsin B is released into the cytoplasm only after activation of the NLRP3 inflammasome [39], the interaction between cathepsin B and NLRP3 occurs outside the lysosome or mitochondria, in a transient manner, and not as part of the inflammasome or binding to ASC or pro-caspase-1 [38].

Cathepsin B can also induce oxidative stress, known in AD, which may allow the interaction of thioredoxin protein (TRX) with NLRP3, and activate it, reinforcing the interaction between the two enzymes that are the objects of studies in neuroinflammation [40].

Studies show that cathepsin B stimulates microglia to release inflammatory mediators, playing a central role in chronic inflammation. Such mediators can induce apoptosis, causing neuronal loss and cognitive decline, in addition to releasing reactive oxygen species, which can also be toxic to neurons [41,42].

Inhibition of cathepsin B in microglia through RNA signaling results in a reduction in Aβ-induced toxic effects [43]. Thus, it is important to discover new inhibitors, preferably reversible, aimed at the treatment of Alzheimer’s disease. Oral administration of E-64d, a potent inhibitor of cathepsin B, in APP/Lon mice improves memory deficits and reduces levels of Aβ(1–40) and Aβ(1–42), as well as truncated peptides pGlu-Aβ(3–40) and pGlu-Aβ(3–42) (pGlu = pyroglutamate). However, such an inhibitor is poorly selective, and inhibits other types of cathepsin as well [16].

Another potent and selective cathepsin B inhibitor, CA-074, when administered as a prodrug (CA-074Me) in mice genetically modified for AD, causes memory improvement and reduces Aβ(1–40) and Aβ(1–42) in the brain [44]. However, both inhibitors are irreversible, and can cause serious adverse effects [45].

Some cathepsin B inhibitor molecules have already been identified in marine invertebrates. Shishicrellastatin A and B, two steroids isolated from the Australian marine sponge *Crella spinulata*, show enzyme inhibition with an IC_50_ value of 8 μg/mL [46]. Another inhibitor was identified from the Pacific sponge *Theonella* aff. *mirabilis*, with an IC_50_ of 29.0 ng/mL [47].

Here, we identified the presence of betaine as a possible inhibitor of cathepsin B by using mass spectrometry. According to PubChem, the molecular formula of betaine is C_5_H_10_NO_2_ and monoisotopic mass 117.07897, with 0.0098 Da difference to the found mass experimental 117.0887 Da.

Betaine (under the forms glycine betaine or proline betaine) has already been described for various species of Cnidaria, such as corals (e.g., *Lobactis scutaria*, *Pocillopora damicornis*, *Pocillopora meandrina*, *Montipora capitata*, *Porites compressa*, *Porites lobata, Australopsammia aurea*), jellyfish (e.g., *Cassiopea andromeda*), and even for the sea anemone *Exaiptasia diaphana* (as *Aiptasia pulchella*), as playing a role in regulating cellular osmotic pressure. It is interesting to notice that all the listed species have symbiotic zooxanthellae in which betaine is also detected—the only exception is the azooxanthellate coral *Australopsammia aurea* (former *Tubastrea aurea*), corroborating the autochthonous production of betaine in cnidarian tissues [48].

Betaine has already been described as an inhibitor of the activity of some enzymes, such as cholinesterase [49]. Studies also show inhibition of cathepsin K by betaine-attenuated osteoarthritis, since this cathepsin is involved in bone resorption and osteoclastogenesis [50].

Furthermore, a study shows that betaine inhibits amyloid peptide aggregation and toxicity caused by transglutaminase and lysyl oxidase enzymes in vitro, demonstrating its potential to decrease fibril formation mediated by extracellular matrix enzymes and oxidative stress [51].

Thus, two enzyme inhibitors, never considered for neuroinflammation, were identified from Brazilian marine invertebrates, and can be considered as prototypes for the treatment of neurodegenerative diseases, such as Alzheimer’s.

## 4. Materials and Methods

### 4.1. Species Studied

The biological material investigated comprised four species of cnidarians (*Chiropsalmus quadrumanus*, *Exaiptasia diaphana*, *Palythoa caribaeorum*, *Renilla reniformis*) and one species of echinoderm (*Lytechinus variegatus*). Marine specimens were collected on São Sebastião Island, São Paulo, Brazil (23°46′23″ S, 45°21′25″ W/23°49′44″ S; 45°25′23″ W/23°49′53″ S; 45°31′18″ W), under Brazilian Environmental Agency (ICMBio/IBAMA) license #16802-2 and #13852-1.

The only planktonic species, *Chiropsalmus quadrumanus*, is a box jellyfish somewhat abundant (Cubozoa) in the tropical western Atlantic, with a complex and severe venom, but little is known about its life history [52,53].

*Exaiptasia diaphana* is a sea anemone (Anthozoa, Actiniaria) distributed worldwide in tropical and subtropical waters up to 30 m deep [54]. The species is widely known by its junior synonym, *Exaiptasia pallida*, a widely used model system for studies on symbiotic relationships with dinoflagellates and cnidarian bleaching under climate change. *Palythoa caribaeorum* is an abundant encrusting colonial zoanthid (Anthozoa, Zoantharia) distributed in intertidal and subtidal areas along the tropical western Atlantic [55], from which is described the well-known palytoxin [56]. The last cnidarian is the sea pansy, *Renilla reniformis* (Anthozoa, Octocorallia), formed by polymorphic colonies inhabiting unconsolidated substrates from the intertidal zone up to mesophotic areas of the tropical western Atlantic [57]. Several sea pens are known for their bioluminescence, and the sea pansy is also known for its production of secondary metabolites used for defense [58].

The green sea urchin, *Lytechinus variegatus* (Echinodermata, Echinoidea), is another abundant species originally from the sublittoral of the tropical western Atlantic [59].

### 4.2. Extracts Attainment

After sampling, animals were washed with filtered sea water and immersed in methanol containing 0.1% acetic acid for 48 h at room temperature. The resulting extract was centrifuged at 5000× *g* for 10 min and the supernatant was lyophilized. The remaining contents were then dissolved in ultrapure water and stored at −20 °C. For the sea urchin *L. variegatus*, the coelomic fluid was obtained by inserting a needle into the peristomal cavity. The liquid was centrifuged at 3000 rpm and the supernatant stored at −20 °C [28,60].

### 4.3. Fractionation

Samples were fractionated by reverse-phase high-performance liquid chromatography (RP-HPLC) in Agilent 1260 Infinity equipment (Agilent, Santa Clara, CA, USA). Aliquots of 80 μL were inserted into the equipment, with a Phenomenex C18 column (4.6 × 250 mm, 300 Å) coupled. Elution was performed by gradient from 0 to 100% B in 30 min, with mobile phase A = 0.1% trifluoroacetic acid in ultrapure water and B = acetonitrile/Milli Q water/0.1% trifluoroacetic acid (900:100:1 *v/v/v*), at a constant flow rate of 1.0 mL/min. The eluted content was monitored by absorbance at 214 nm and the peaks were manually collected, according to the chromatogram obtained. In the case of re-fractionation, the same equipment was used, but with a C8 column (4.6 × 150 mm, 300 Å), with a constant flow of 1.0 mL/min and isocratic elution, using 0.1% trifluoroacetic acid in ultrapure water. Detection was also performed by measuring the absorbance at 214 nm.

### 4.4. Mass Spectrometry

Fractions collected after HPLC were analyzed by mass spectrometry (Xevo GS QToF, Waters Co., Milford, MA, USA). Aliquots of 5 μL were inserted into the equipment, without a chromatographic column. Elution was performed with 50% acetonitrile containing 0.1% formic acid. The equipment was set in positive ESI ionization mode and data were collected in a full MS scan ranging from 100 to 1000 *m/z* in high and low voltage. Raw data were analyzed by Progenesis QI software (Waters Co., Milford, MA, USA) to identify low mass compounds by means of comparison with GNPS or Vaniya/Fiehn Natural Products database, both with tolerance of up to 5 ppm for the precursor.

### 4.5. Enzymatic Assays

Assays on caspase-1 were conducted in 384-well plates, in triplicate, with a final reaction volume of 20 μL using the Caspase-1 Inhibitor Screening Assay Kit (Cayman Chemical, Ann Arbor, MI, USA). Samples (10 μg) were previously incubated with enzymes (recombinant human caspase-1) treated with 2 mM DTT for 10 min at room temperature. Buffer was added to the wells and then the synthetic substrate (Ac-VEID-AFC, 4 μM). A known caspase-1 inhibitor, Z-VEID-CHO (10 µM, Cayman Chemical), was used as a positive control. The increase in fluorescence after incubation was measured by a fluorimeter at the excitation wavelength of 400 nm and emission wavelength of 505 nm (GloMax^®^, Promega, Madison, WI, USA). All fluorescence values were subtracted from the background (buffer).

Cathepsin B assays were performed in 384-well plates, in triplicate, with a final reaction volume of 20 μL, using the Cathepsin B Inhibitor Screening Kit (Sigma-Aldrich, St. Louis, MO, USA). Samples (10 μg) were previously incubated with enzymes (recombinant human cathepsin B) treated with 2 mM DTT for 10 min at room temperature. Buffer was added and then 10 mM of synthetic substrate (Ac-RR-AFC). A known inhibitor, FFFMK (10 μM), was used as a positive control. The increase in fluorescence 50 min after incubation was measured by a fluorimeter at the excitation wavelength of 400 nm and emission wavelength of 505 nm [61]. Velocity was calculated as ∆y (AUF)/∆x (time).

## 5. Conclusions

We obtained two molecules with enzymatic inhibitory activity, viz. trigonelline for caspase-1, from the box jellyfish *C. quadrumanus*, and betaine for cathepsin B, from the sea anemone *E. diaphana*. These molecules represent alternatives for the control of neuroinflammation, and may become important therapeutic targets, and may contribute to the treatment of neurodegenerative diseases

## Data Availability

Publicly available datasets were analyzed in this study. This data can be found at www.inovamol.com.br.
